# Cognitive interventions for memory and psychological well-being in aging and dementias

**DOI:** 10.3389/fpsyg.2023.1070012

**Published:** 2023-02-02

**Authors:** Cassandra J. Dinius, Carmen E. Pocknell, Michelle P. Caffrey, Richard A. P. Roche

**Affiliations:** Department of Psychology, Faculty of Science and Engineering, Maynooth University, Co Kildare, Ireland

**Keywords:** memory, aging, dementia, reminiscence therapy, cognitive reserve, lifestyle, non-pharmacological interventions

## Abstract

The human lifespan has expanded drastically in the last few centuries, due to improvements in sanitation, medicine, and nutrition, but with this increase in longevity comes higher rates of cognitive pathology such as mild cognitive impairment (MCI) and dementia; the latter is estimated to reach more than 75 million people by 2030. Pathology risk is related to measures of executive function, lifestyle factors (e.g., education, occupation, and leisure activities), and cognitive reserve. One way of building cognitive reserve may be to structure the environment to encourage lifelong engagement and learning, and since a pharmacological “cure” for dementia remains elusive, non-pharmacological approaches such as physical activity, social engagement, and cognitive stimulation are becoming increasingly essential to preserving and protecting brain health. Here, we describe our recent research into Reminiscence Therapy (RT) to promote cognitive and psychological function in old age and early dementia. We review the Recall Initiative, which involved brain imaging and behavioral indices of memory pre- and post-RT. We also report results from a pilot study—AIM WARM—in which RT was combined with physical activity, specifically walking, for early-stage dementia. Finally, we outline our future directions for tailored reminiscence interventions in combination with other activities (e.g., yoga and meditation) for different groups, namely early Alzheimer’s disease, Semantic Dementia, and older individuals in the prison system.

## 1. Introduction

The human lifespan has expanded drastically in the last few centuries, due to improvements in sanitation, medicine, and nutrition (Olshansky et al., 2005). This should not imply that a longer lifespan is accordingly free of disease or disability; rather rates of physical and cognitive pathology are also rising (Livingston et al., 2020). Some age-related cognitive change is normal and occurs in the absence of disease. Older adults can expect slowed processing speed, diffuse attention, and difficulty learning new information (Birren et al., 2006; Craik and Salthouse, 2011). These normal age-related changes are distinct from cognitive pathology, including neurocognitive disorders like mild cognitive impairment (MCI) and dementia (Cabeza et al., 2018).

Mild cognitive impairment (MCI) and, to a greater extent, Alzheimer’s’ Disease and Related Dementias (ADRD) are classified by changes in cognitive performance, which may impact realms like planning, memory, learning, and attentiveness (Turner et al., 2020). In MCI, these changes are subtle and may not significantly impact activities of daily living or the ability to live independently. A diagnosis of MCI increases the likelihood of converting to dementia, but not all cases are prodromal (Cabeza et al., 2018).

The term dementia refers to a group of progressive changes that are marked by neurodegeneration in specific brain regions. Accordingly, downstream changes can be observed in domains like executive function, perceptual-motor abilities, and personality (Turner et al., 2020). People living with dementia experience significant and persistent changes in cognition, but these are not immediately fatal. Instead, the long-term outcomes are loss of independence and continued declines in cognitive ability. The progressive nature of dementia, combined with improved medical advances for comorbid diagnoses, means that people are living longer with this diagnosis (Livingston et al., 2020). As such, dementia impacts social and economic planning at every level including housing, health, urban and rural planning, transport, policing, and the business environment. Dementia is wide-reaching, estimated to impact more than 80 million people by 2030, and over 150 million worldwide by 2050 (Nichols et al., 2022).

Alzheimer’s disease (AD) represents 60–70% of dementia diagnoses and is subsequently the most common form of dementia (Rizzi et al., 2014). The pathophysiology of AD is, in part, believed to be connected to beta-amyloid peptide (Aβ) deposition throughout the brain (Sepulcre et al., 2018). It has emerged that one of the cornerstone beliefs about the biochemical basis of AD—the presence of Aβ plaques in the brain—may contain fabricated data, potentially undermining decades of research into chemical-based interventions (Lesné et al., 2006). The treatment for AD remains elusive, and available treatments provide a mere stopgap in the form of relief of symptoms from cognitive decline (Srivastava et al., 2021). A discovery to cure dementia is unforeseen and those living with Alzheimer’s are left waiting. A reliable avenue of research may be the impact of lifestyle factors, which have shown promising results.

## 2. Non-pharmacological approaches

Health and longevity are impacted by innumerable factors ranging from how often someone flosses their teeth to how they spend their leisure time (Scarmeas and Stern, 2003; Brown, 2019). Risk factors that increase the likelihood of dementia include aspects of the general environment (e.g., geography, sun exposure, and air pollution) as well as the habits of individuals (e.g., participation in leisure activities, psychological well-being, and age at retirement).

The latter, lifestyle-based risk factors, encompasses a variety of modifiable variables. In the absence of a cure, this presents an opportunity to examine how lifestyle-based factors may be addressed to influence dementia risk. The risk of developing dementia is shaped by several lifestyle factors, including levels of physical activity, social engagement, mental well-being, and cognitive stimulation (Reuter-Lorenz and Park, 2010). These elements of daily living can be used to predict the risk of receiving a dementia diagnosis as well as forecasting the rate of decline (Deckers et al., 2019).

Accordingly, this knowledge about risk factors can be used to mitigate dementia diagnoses. Due to the high social and financial costs of dementia, delaying a diagnosis is a viable strategy for burden reduction. By addressing the risks and recognizing the roles of vulnerability and prevention, rates of dementia could drop by 40% (Livingston et al., 2020). Lifestyle factors that decrease the risk of dementia include moderate physical activity, psychological well-being (e.g., no diagnosis of depression), and regular social engagement (Livingston et al., 2020; Sikkes et al., 2021). These are all modifiable factors that can be integrated into interventions using a non-pharmacological approach.

Non-pharmacological approaches are generally easily adopted, have few side effects, and can be combined with pharmacological treatments without interference (Sikkes et al., 2021). As such, these approaches can complement people with complex care needs or those living in institutional settings with a high care burden. Thus, non-pharmacological approaches are feasible for intervention studies and can be used with a variety of populations. In the following section, we describe some non-pharmacological approaches.

### 2.1. Cognitive reserve and engagement

As a pharmacological “cure” for dementia remains unforeseen, it becomes critical that we utilize non-pharmacological approaches to maximize brain health and resiliency. While the causal understanding of dementia pathology is yet unknown, there is evidence for protective effects (Li et al., 2016). Randomized clinical trials (RCT) of physical exercise demonstrate robust protection of cognition (Ahlskog et al., 2011). Complementing this experimental work are epidemiological and correlative studies. Here, research suggests that psychological well-being and social engagement are not only protective of cognition but also can reduce dementia risk altogether (Wilson et al., 2003; Park et al., 2007).

We rely on the knowledge that modifiable lifestyle factors like physical exercise, psychological well-being, and social engagement can preserve and protect brain health. How might these lifestyle factors influence cognition and buffer against pathology? Researchers are only beginning to understand the mechanisms that subserve age-related resilience in cognitive efficiency. Possibilities include improvements in basic cognitive abilities, the development of strategies, and the automatization of selective aspects of a skill or task (Baltes et al., 1999). Cabeza and colleagues suggested that high-performing older adults may be compensating for neural decline by reorganizing neurocognitive networks (Cabeza et al., 2002). This suggestion of “neural plasticity” led to many theories that sought to answer the question: does the aging brain attempt to compensate for cognitive change, and how might the brain achieve this goal?

### 2.2. Cognitive resources and compensation

The need for compensation can be predicted by lifestyle factors that influence the health and plasticity of the brain. Protective “neural resource enrichment” (e.g., participating in exercise, leisure, and social activities) may preserve or enhance brain function. On the other hand, antagonistic “neural resource depletion” may accelerate age-related declines in performance (e.g., APOE-4 gene, smoking, and obesity; Reuter-Lorenz and Park, 2014). Again, we highlight the role of modifiable factors in the aging brain by recognizing that compensation can be helped or harmed by lifestyle factors.

The notion of “brain maintenance” suggests that healthy genetics and lifestyle factors may result in a need for less compensation (Reuter-Lorenz and Park, 2014) see Nyberg et al. (2012) for more on brain maintenance. The resources that are drawn upon during the act of compensation are built and maintained, perhaps throughout the lifespan (Cabeza et al., 2018). By providing opportunities for neural enhancement, the brain can counter age-related decline and other insults. That is, by maintaining resources and building a reserve, the aging brain can effectively buffer decline (Burke et al., 2019).

Though the occurrence of cognitive aging is well-documented, there is less empirical support concerning the mechanisms behind this process and where in the nervous system, it may originate (Salthouse, 2004). Neuroimaging allows for the observation of brain processes by revealing a window into the neuroanatomical substrates of cognitive functions (Festini et al., 2018). Various theories and models (Hemispheric Asymmetry Reduction in Older Adults; HAROLD; Cabeza et al., 2002); Posterior–Anterior Shift in Aging (PASA; Grady et al., 1994, 2003); and Compensation-Related Utilization of Neural Circuits Hypothesis (CRUNCH; Reuter-Lorenz and Cappell, 2008) attempt to describe differential processing patterns that are observed in young versus older brains. Perhaps, older adults are compensating for neural decline by reorganizing neurocognitive networks (Grady et al., 1994, 2003; Cabeza et al., 2002; Reuter-Lorenz and Cappell, 2008; Festini et al., 2018).

During verbal recall tasks, older adults who demonstrate network activation similar to young adults (e.g., right prefrontal cortex) perform poorly, whereas the highest performing older adults demonstrate unique activation patterns (e.g., bilateral prefrontal cortex; Cabeza et al., 2002). Furthermore, adults demonstrate stronger anterior activation relative to young adults, but weaker posterior (e.g., occipital) activation relative to young adults (Grady et al., 1994, 2003). These patterns of overactivation (which may include the prefrontal cortex as seen in Grady et al., 2003 or bilateral as seen in Cabeza et al., 2002) relative to young adults exist under conditions of low cognitive demand. As cognitive demand conditions increase and tasks become more challenging, older adults “max out” their resources and display under-activation and subsequent decline in performance (Reuter-Lorenz and Cappell, 2008).

Cognitive changes are typical across the lifespan, though here we focus instead on pathological forms of decline. Age-related declines in function may be addressed *via* compensation, perhaps in the form of “scaffolding” (Reuter-Lorenz and Park, 2014). Compensation refers to the ability of the brain to increase activation or recruit additional resources to cope with heightened demand (Cabeza et al., 2018). The process of compensation may be assisted by scaffolding, which can be internal (e.g., rerouting neural networks or increasing neural activation) and external (e.g., environmental cues and strategies; Reuter-Lorenz and Park, 2014). New learning, cardiovascular health, and certain types of cognitive training may enhance the brain’s ability to procure effective scaffolding (Reuter-Lorenz and Park, 2014).

Reserve is a concept that may explain the heterogeneity associated with susceptibility to dementia. It may also be used to describe varying rates of change seen in both the normal and pathological aging process (Schneider and Pichora-Fuller, 2000). Aging is associated with a normal decline in a multitude of cognitive processes and brain functions. Yet some people display relatively minor intraindividual loss of function while others exhibit steep declines in their rate of change (Stern, 2012). So too in pathological aging, the rate of change and trajectory of disease can vary widely.

These pathological trajectories can be described both in terms of interindividual and intraindividual change. For example, the time between a dementia diagnosis and loss of independence can be relatively quick, while others live with the diagnosis for decades (Jagust, 2018). Exhibiting interindividual change, a person may exhibit preservation of function for many years, followed by a precipitous decline (Walsh et al., 2022). Understanding the rate of change and trajectory of decline is critical for maintaining activities of daily living, and thus independence (Hertzog et al., 2008; Walsh et al., 2022). Given a diagnosis of dementia, the ability to retain function presents an important leverage point in the disease. Cognitive reserve may flatten the slope of decline and contribute to preserved independence (Song et al., 2021).

A growing body of literature suggests that age-related decline in cognition can sometimes be reduced through interventions of physical activity, social engagement, and mental well-being (Erickson et al., 2011; Gajewski & Falkenstein, 2016; Allen et al., 2020). These interventions utilize lifestyle factors that are known promoters of cognitive reserve. High levels of cognitive reserve may decrease the risk of dementia by as much as 46%, relative to those with moderate to low levels of cognitive reserve (Stern, 2012). Reserve can be classified as either “brain” [e.g., quantitative measure of neurons or brain-derived neurotrophic factor (BDNF)] or “cognitive” (e.g., interindividual differences in performing mental tasks that enable resilience to brain insult, which can be drawn upon during the act of compensation, see the previous section; Stern, 2012). The delineation between brain and cognitive reserve is under debate, with a proposed move toward referring to general “reserve” rather than these separate aspects (Cabeza et al., 2018).

It follows, then, that building cognitive reserve, or enhancing resilience against cognitive insult, is a crucial component of non-pharmacological approaches to promoting cognitive and psychological function in old age and early dementia. Correlational studies between lifestyle variables and cognitive performance show protective benefits (cognitive flexibility, delayed dementia diagnosis, and increased independent living; Jaeggi et al., 2020). Physical activity, social engagement, and cognitive stimulation are all suitable candidates for intervention-based work that reduce the likelihood of dementia and cognitive decline. An important goal for future research is to determine when benefits are and are not produced.

### 2.3. Cognitive training

Cognitive training studies often demonstrate brain-volumetric increases and increased activation patterns in targeted regions (Lustig et al., 2009). Strategy-based training can demonstrate benefits on the specific task (near-transfer) but lacks benefits on general performance (far-transfer; Lustig et al., 2009). Cognitive performance shows an immediate benefit from training (increased cognition in 86% of the speed of processing group, 74% of the reasoning group, and 26% of the memory group; Gross and Rebok, 2011). However, performance increases on training effects are highly specific. For example, training on memory can increase performance on memory tasks but not speed of processing or reasoning (Gross and Rebok, 2011). Engagement-based interventions can improve cognition and performance while demonstrating far-transfer benefits on a range of tasks.

The engagement model strives to embed the participant in complex environments that are socially enriching (Stine-Morrow et al., 2014). Engaging in social and intellectual activities may buffer age-related declines in cognition even without explicit instruction (Stine-Morrow et al., 2014). One explanation for this benefit is that both social and intellectual challenges require a diverse range of abilities rather than mastery of one skill (e.g., being a Bridge player requires social skills, mathematical abilities, and memory—being a maths prodigy alone will not allow you to master the game). Non-pharmacological interventions that demand cognitive engagement will draw on various skill sets and require cognitive flexibility (Park et al., 2007; Stine-Morrow et al., 2007, 2014; Parisi et al., 2009). The Synapse project is one example of a robust study that examined this association through randomized controlled trials to high- (e.g., learning digital photography) and low- (e.g., trivia or active listening) engagement conditions. Those who were assigned to the high-engagement condition exhibited improvements in neural function and episodic memory. These high-engagement skills may promote the use of distributed networks and promote neural efficiency, thus increasing strategy and scaffolding (McDonough et al., 2015). This may be a mechanism by which various abilities could then contribute to building cognitive reserve (Stine-Morrow et al., 2008).

Group-based interventions foster social interaction, often in the presence of learning a new skill or navigating a novel environment. This approach may boost engagement by requiring the participants to remain socially active while also learning or practicing novel tasks. Some of these studies show benefits for older adults and also may delay dementia (Jaeggi et al., 2014). Perhaps, the building of cognitive reserve is a contributing factor in conferring protective benefits.

Non-pharmacological approaches offer a promising avenue for modifying dementia risk. These approaches often target lifestyle-based factors like moderate physical activity, psychological well-being, and regular social engagement. Interventions which target these factors can demonstrate cognitive protection against dementia, allowing older adults to experience an increased quality of life with greater autonomy and independence. The mechanism for this protection may be explained by the concept of cognitive reserve, which supports a buffer against cognitive insult.

### 2.4. Reminiscence therapy

Reminiscence Therapy (RT) is now a popular treatment for depression, loneliness, and anxiety in older adults by dealing with past life experiences (Hsieh and Wang, 2003; Scogin et al., 2005; Woods et al., 2005; Chueh and Chang, 2014; Lopes et al., 2016; Syed Elias et al., 2019). Structured RT, where triggers and prompts are provided for discussion in a group setting, seems to increase psychological well-being such as life satisfaction, communication, and self-esteem (Baines et al., 1987; Chao et al., 2006; Yamagami et al., 2007; Preschl et al., 2011; Zhang et al., 2015; Allen et al., 2018). Guided reminiscence provides an opportunity for participants to recall stories and anecdotes, while a trained facilitator leads the group and guides the reminiscence topics. Interestingly, RT appears to have a positive effect on global cognition as well in dementia patients who report improved mood, well-being, and cognitive function after 4–6 weeks (Woods et al., 2005; Wang, 2007; Azcurra, 2012; Lopes et al., 2016).

Most notably, RT appears to have therapeutic benefits, particularly for dementia patients (Coll-Planas et al., 2017). Both group-based and individual RT encourage social interaction and cooperation, thus minimizing isolation and depressive symptoms (Coll-Planas et al., 2017; Berntsen and Kirk, 2019). In dementia patients, RT may allow for rehearsal of conversational skills, as well as inspiring a renewed sense of purpose and interest in the world (Baines et al., 1987). Furthermore, RT leads to improved mood, with positive reminiscences evoking generally positive feelings, which may be sustained and augmented with increased sessions (Cappeliez et al., 2008; Coll-Planas et al., 2017). In particular, there is evidence to suggest that triggering reminiscence with music encourages positive associations, thus improving mood and, potentially, cognition (Engelbrecht et al., 2021).

Though the majority of studies support RT as a mood-boosting mechanism primarily, thus impacting cognition indirectly by improving emotional state and increasing motivation and general well-being, there is some evidence to support neurological changes associated with RT. Research demonstrates increased blood flow in the frontal lobe as well as reduced neuroimaging abnormalities associated with RT (Tanaka et al., 2007). Furthermore, reminiscing while listening to music results in greater frontal and central bilateral activation, suggesting that RT, particularly in conjunction with a mediating variable such as music, may have a direct impact on cognition (Engelbrecht et al., 2022). It is possible that the act of reminiscing itself may also be beneficial to global cognition. Consistent, prompted retrieval encourages reactivation and reinforces existing memory traces, as well as allowing for the formation of new associations, particularly when there is a lag between reminiscence sessions (Delaney et al., 2010).

In recent years, research has pointed to the benefits to both mental health and cognitive performance (e.g., memory, attention) of physical exercise and, separately, of actively engaging in guided use of one’s memory, for example through Reminiscence Therapy (RT). These benefits appear to apply to both healthy older adults and those in the early stages of conditions associated with memory problems, such as dementias including Alzheimer’s disease (AD). Evidence suggests that physical activity/aerobic exercise (see Gomez-Pinilla and Hillman, 2013)— and in particular walking (see O’Mara, 2019)—results in an array of cognitive and psychological benefits which are underpinned by structural and functional changes in memory-related brain structures, particularly the prefrontal cortices and hippocampus.

A growing literature has begun to suggest that such reminiscence activities can lead to psychological and/or cognitive gains for healthy, community-dwelling older individuals as well as those living with dementias. Other studies suggest that the benefits of RT can be enhanced by combining it with other interventions such as exercise or creative engagement. This, primarily, is what makes RT so unique as an intervention; combining RT with other activities allows for a more tailored, bespoke intervention that is better suited to the individual. Moreover, these interventions are non-invasive, stimulating, and enjoyable, impacting and often improving quality of life (Berntsen and Kirk, 2019).

### 2.5. Exercise

In the older population, the task of finding solutions to cognitive decline is still a major challenge. One powerful potential avenue is exercise, which has shown promising effects on cognitive abilities and general quality of life in older adults (Gomez-Pinilla and Hillman, 2013). Neuroimaging has also demonstrated exercise’s impact on functional brain plasticity (Cui et al., 2018). Increasingly frequently, public health guidelines advocate exercise as an essential tool for the prevention and/or moderation of the number of dementia cases worldwide (Erickson et al., 2011; Gajewski and Falkenstein, 2016).

By definition, exercise is “*a type of physical activity consisting of planned, structured, and repetitive bodily movement done to improve and/or maintain one or more components of physical fitness*” (Caspersen et al., 1985).

Unfortunately, the decrease in human physical capacities is often associated with the process of aging and tends to go hand-in-hand with a decline in an individual’s activity levels. Remarkably, exercise appears to offer a protective function, or at least helps to preserve global cognition and human functional capacity such as muscle mass or cardiorespiratory function. More precisely, aerobic and resistance exercises are shown to have strong effects on general health, such as improved endocrine function or better cognitive abilities (Valenzuela et al., 2019). In contrast with aerobic activities, only a small quantity of resistance exercise is actually needed to show benefits on cognition for older adults, and particularly on memory (Sun et al., 2021). This effect was explained by the essential value that resistance training can have on an individual’s quality of life for people living with dementia (Erickson et al., 2011; Barha et al., 2017).

In fact, various studies have shown that the combination of multiple non-pharmacological activities, rather than one, has stronger outcomes for preventing global cognitive/executive functional decline in people living with Mild Cognitive Impairment (MCI). There has also been a sharp increase in literature promoting the benefits of multicomponent exercise interventions in helping the global well-being and cognition of older adults (Paramos-de-Carvalho et al., 2021).

The World Health Organization (WHO), in October 2022, highlighted the importance of the older population committing to at least 150–300 min of moderate-intensity aerobic exercise every week or, alternatively, 75–150 min of vigorous aerobic exercise of muscle strengthening activities (including the major muscle groups) for a minimum of three times per week. However, there remains no real consensus on the minimum quantity needed to show benefits on cognition, reflecting the ideas that for the older population, “doing some physical activity is better than doing none” (Bull et al., 2020), and that “the shorter and more frequent the better” (Sanders et al., 2019).

Globally, exercise is one of the most reliable interventions demonstrated to have real effect on the prevention/treatment of cognitive decline/dementia in older adults. However, it is still ambiguous which type of exercise is the most reliable and, more importantly, which exercise is the best for each population. However, a recent review paper has suggested that low/moderate levels of aerobic exercise over medium to long durations (e.g., walking, cycling) may be more beneficial for memory for young and older adults in the pre-encoding phase, while comparable levels of anaerobic/resistance exercise may be more effective post-encoding, and less so for older adults (Loprinzi et al., 2021). This distinction warrants further exploration. Aerobic fitness has been identified as a preventative measure with regard to natural hippocampal degradation; higher aerobic fitness is associated with larger hippocampal volume and improved memory in older adults (Erickson et al., 2011). This may be a result of the similarities between aerobic exercise and successful encoding of memories—both require repetition. It is likely that aerobic exercise carried out in conjunction with RT may be particularly beneficial, given the effect of each on the hippocampus.

The findings of our projects (*see below*) will focus on understanding and measuring the impact of different types of exercises. By doing so, we hope to address one of the major health and social concerns of the 21st century.

## 3. Interventions

### 3.1. Recall

Next, we review the Recall Initiative, which involved brain imaging and behavioral indices of memory pre- and post-RT. In 2019, we established a 2-year project, the RECALL Initiative, funded by the IRC New Horizons funding scheme. We focused on the potential benefits of reminiscence therapy on memory and well-being for healthy older adults living in the community, and people in the early stages of dementia. The initial study incorporated pre- and post-reminiscence testing of processes including memory, attention, and executive function, as well as psychological variables—anxiety, depression, quality of life, and social engagement. A small sub-group also took part in functional MRI brain scanning at Trinity College Institute of Neuroscience to test for structural and functional brain changes as a result of the intervention. A Philips Intera Achieva 3.0 T MR system (Best, The Netherlands) was used to acquire the MRI data. Preprocessing was performed using fmriprep^1^ and fMRI statistical analyses were performed with SPM12 (Statistical Parametric Mapping; Wellcome Trust Center for Neuroimaging, London, United Kingdom).^2^ During the group sessions, we recorded audio (with permission) of the stories and memories that our participants recalled, with these recordings hosted in a designated section of the Digital Repository of Ireland.^2^

**Figure 1:**
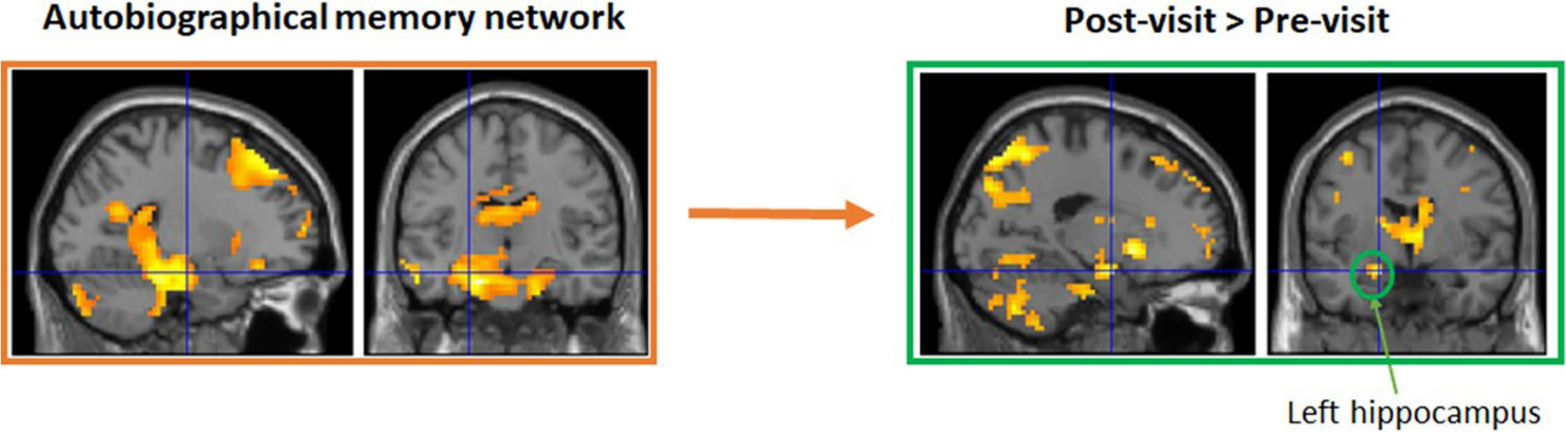
A small sub-group also took part in functional MRI brain scanning at Trinity College Institute of Neuroscience to test for structural and functional brain changes as a result of the intervention. Functional imaging data are currently being analyzed (Viard et al., in preparation). Initial results suggest the classical autobiographical memory network (including the precuneus, medial frontal and lateral temporal gyri, and hippocampi) was activated for pre- and post-visits separately. Direct comparisons showed greater activation for post-visit in left superior medial frontal gyrus, precuneus, and anterior hippocampus, compared to pre-visit.

Groups in Maynooth, Swords, Tallaght, and Dublin city center all took part in 6 weeks of reminiscence. Weekly groups recalled stories on their earlier years about personal experiences and major national events, with the help of photos, such as the visit of John F Kennedy in 1963 and the Pope in 1979. While only modest improvements were observed in these groups, this is likely due to the fact that they were a cognitively very healthy group at the outset and the fact that numbers were low in each group, reducing statistical power (Allen et al., 2020, 2021a). To examine potential benefits for cognitive decline, a modified version of this intervention was administered to a small group of older adults living with dementia, with a weekly group meeting in Peamount Healthcare’s Dementia Unit. Again, measures of cognition and psychological health were taken before and after 6 weeks of reminiscence, with some indications of improvements in certain aspects of memory (Allen et al., 2021b). An additional patient experiencing Transient Epileptic Amnesia (TEA) also participated in the intervention, and again some modest improvements were observed. Functional imaging data are currently being analyzed (Viard et al., in preparation). The classical autobiographical memory network (including the precuneus, medial frontal and lateral temporal gyri, and hippocampi) was activated for pre- and post-visits separately. Direct comparisons showed greater activation for post-visit in left superior medial frontal gyrus, precuneus, and anterior hippocampus, compared to pre-visit (see Figure 1).

The social interaction element of the intervention appears to be a key mechanism driving any changes observed; by encouraging healthy older adults and people living with dementia to engage in a weekly, semi-structured, peer-to-peer group activity, social as well as cognitive function are engaged, leading to a greater likelihood of engagement, reduced dropout, and a more positive overall experience for participants.

### 3.2. AIM-WARM

In our Age-Inclusive Maynooth: Walk and Recall Memories (AIM-WARM) work, which was funded by an Irish Research Council New Foundations grant 2020, we investigated the potential impact of combining the exercise of walking and reminiscence therapy, in early Alzheimer’s disease groups. We measured the potential benefits of this combination on autobiographical memory, cognitive abilities, and psychological well-being in a small-sample pilot study (*n* = 2).

Performance on cognitive and psychological measures was taken pre- and post-intervention, on paper, and in individual sessions with each participant.

Cognitive measures were assessed with the Montreal Cognitive Assessment (MoCA, Nasreddine et al., 2005) and the Episodic Autobiographical Memory Interview (EAMI; Irish et al., 2008). Psychological well-being data were measured with the help of the Control, Autonomy, Self-Realization, and Pleasure Quality of Life Questionnaire (CASP-19; Netuveli et al., 2006); the Satisfaction with Life Scale (SWLS; Diener et al., 1985); and lastly the Holden Communication Scale (Strøm et al., 2016).

We again employed the Patient and Public Involvement (PPI) approach, which was established in our previous work and will continue to be employed in each of our future projects. Details of this approach can be found here.^4^ Four PPI consultants/advisors (two people living with dementia and two carers) were recruited through the Dementia Research Advisory Team, from the Alzheimer’s Society of Ireland (two women, two men), and were meticulously embedded throughout the project—from the design to interpretation/dissemination of the results. Their contributions strongly improved the content of the intervention and taught us the most effective, applicable, and convenient method to employ in this study.

With assistance from the geography department, we planned a geographical tour including specific historical sites as visual cues, selecting those that may be most familiar to the community participants (i.e., local church). Prior to the experiment, care was taken to complete a Walkability Audit Tool for Roads and Streets, to make sure that the routes were safe. Additionally and as mentioned, people with dementia and carers also offered unique insights and viewpoints to improve our research project, including for these walking-routes. Lastly, our selection criteria included people who were familiar with, or who used to live in Maynooth Town, for more engaged reminiscence sessions. The intervention group completed hourly, twice-weekly sessions for 2 weeks, walking and reminiscence-based interventions. During the intervention, we stopped to discuss each historical site/personal memories, and recorded the conversations for later anonymized upload to the Digital Repository of Ireland (DRI) archive.

Autobiographical Memory was one of the main focuses within the study. In the intervention group, after the completion of four reminiscence-plus-walking sessions, no significant main effect was found, despite an interesting increase in semantic memory (i.e., the name of a friend), precisely during the period of middle adulthood (Episodic Autobiographical Memory Interview, EAMI; Irish et al., 2008). This supports the reminiscence peak between 10 and 30 years of age. In parallel, this also accompanies the Multiple Trace Theory, which supports the possible distinction between semantic and episodic memory (Roche et al., in preparation; data available on Open Science at doi 10.17605/OSF.IO/Q9VAY). Combining reminiscence with walking may represent a viable means to enhance the benefits of these interventions. However, as this is a pilot study, findings should be interpreted cautiously, with further work needed.

Our next project, entitled “Tailored Reminiscence Interventions for Aging and Dementias in Community Settings (TRIADICS),” continues work on tailored, group-based interventions, using larger samples and different populations of interest. This will enable further interpretation and to understand better the potential benefits of these combined interventions.

## 4. Discussion

### 4.1. Future directions

Results from the AIM-WARM initiative will shape the future of our next project, TRIADICS. This project, funded by IRC Coalesce, will assess the benefits to cognition and psychological health using tailored, co-created interventions. These may include exercise, meditation, breathwork, and group reminiscence meetings for older adults and people living with dementias, including Alzheimer’s disease and Semantic Dementia.

We will continue to call on a Patient and Public Involvement (PPI) approach to co-create these tailored therapeutic reminiscence interventions with representatives from each of the four groups of interest: community-dwelling older adults, people living with Alzheimer’s disease, people living with Semantic Dementia, and older people living in prisons. This project will also utilize functional MRI to obtain neuroanatomical data to provide insight into relevant brain mechanisms and structures that may be engaged in reminiscence therapy and throughout this intervention.

Using an iterative process, these Reminiscence Therapy interventions are being refined with the intent to deploy this service on a larger, public scale and disseminate this approach to the broader public. The project will develop, refine, and pilot-test a set of tailored interventions and then validate them through a series of pre-post mixed-methods studies. Subsequently, this regime can be rolled-out in larger-scale initiatives *via* links with Age-Friendly Ireland (AFI) and Meath County Council, the national contact point for age-friendly communities in Ireland. Finally, recalled memories and stories elicited during the program will be recorded, archived, anonymized, and made available to the public as part of our existing, bespoke section of the Digital Repository of Ireland (DRI).

## 5. Conclusion

Brain disease remains a significant societal challenge for the 21st century. The annual cost of brain disease in the EU is estimated at approximately €800 billion, with dementia accounting for over €100 billion of this figure (€105,163,000; (Gustavsson et al., 2011)). This substantial economic cost is mirrored by the catastrophic personal impact dementias have on sufferers and their families/carers, with AD alone affecting some 35 million people worldwide (Ising et al., 2015); this figure is expected to treble by 2050. Any interventions which can effect even a modest reduction in the prevalence or impact of dementia and normal age-related memory decline will lead to substantive personal, social, and financial benefits to society at large, and will reduce strain on already laboring healthcare systems and overworked medical professionals. Lifestyle-based interventions which may bolster resilience and cognitive reserve, while also addressing a need for community engagement and social contact, represent an encouraging avenue for future research, and may present an affordable, enjoyable, and effective means to preserve cognitive function and health into later life in both normality and pathology. Driven by the ethos of PPI, projects such as the Recall Initiative, AIM WARM, and TRIADICS represent our first steps in this direction, and will hopefully lead to novel, creative, and widely applicable interventions for brain health.

## Author contributions

CD, CP, MC, and RR contributed equally to the writing of the manuscript. CD and CP also collected data and carried out PPI workshops for projects reported above for the AIM WARM and TRIADICS projects. RR was PI for all of the above-reported projects. All authors contributed to the article and approved the submitted version.

## Funding

This work was funded by three awards from the Irish Research Council (IRC), specifically IRC New Foundations 2020 (Ref: IRC/NF/2019/ROCHE), IRC New Horizons 2016 (Ref: REPRO/2016/76), and IRC Coalesce 2021 (REF: COALESCE/2021/101) schemes. CD and MC are supported by Coalesce funding, and CP was funded under the New Foundations scheme.

## Conflict of interest

The authors declare that the research was conducted in the absence of any commercial or financial relationships that could be construed as a potential conflict of interest.

## Publisher’s note

All claims expressed in this article are solely those of the authors and do not necessarily represent those of their affiliated organizations, or those of the publisher, the editors and the reviewers. Any product that may be evaluated in this article, or claim that may be made by its manufacturer, is not guaranteed or endorsed by the publisher.

## Footnotes

^1^\https://fmriprep.org/en/stable

^2^\https://www.fil.ion.ucl.ac.uk/spm

^3^\https://repository.dri.ie/catalog/wp98p078n

^4^\https://www.ideasinall.com/walk-me-back-reminiscence-and-physical-activity-for-better-memory/
